# Fluid overload in patients with septic shock and lactate clearance as a therapeutic goal: a retrospective cohort study

**DOI:** 10.5935/0103-507X.20200015

**Published:** 2020

**Authors:** Carmelo José Espinosa-Almanza, Oscar Sanabria-Rodríguez, Iván Riaño-Forero, Esteban Toro-Trujillo

**Affiliations:** 1 Hospital Universitario San Ignacio, Faculdade de Medicina, Pontificia Universidad Javeriana - Bogotá, Colombia.

**Keywords:** Fluid therapy, Edema, Shock, septic, Resuscitation, Lactic acid, Mortality, Hidratação, Edema, Choque séptico, Ressuscitação, Ácido lático, Mortalidade

## Abstract

**Objective:**

To assess whether fluid overload in fluid therapy is a prognostic factor for patients with septic shock when adjusted for lactate clearance goals.

**Methods:**

This was a retrospective cohort study conducted at a level IV care hospital in Bogotá, Colombia. A cohort of patients with septic shock was assembled. Their characteristics and fluid balance were documented. The patients were stratified by exposure levels according to the magnitude of fluid overload by body weight after 24 hours of therapy. Mortality was determined at 30 days, and an unconditional logistic regression model was created, adjusting for confounders. The statistical significance was established at p ≤ 0.05.

**Results:**

There were 213 patients with septic shock, and 60.8% had a lactate clearance ≥ 50% after treatment. Ninety-seven (46%) patients developed fluid overload ≥ 5%, and only 30 (13%) developed overload ≥ 10%. Patients exhibiting fluid overload ≥ 5% received an average of 6227mL of crystalloids (SD ± 5838mL) in 24 hours, compared to 3978mL (SD ± 3728mL) among unexposed patients (p = 0.000). The patients who developed fluid overload were treated with mechanical ventilation (70.7% versus 50.8%) (p = 0.003), albumin (74.7% versus 55.2%) (p = 0.003) and corticosteroids (53.5% versus 35.0%) (p = 0.006) more frequently than those who did not develop fluid overload. In the multivariable analysis, cumulative fluid balance was not associated with mortality (OR 1.03; 95%CI 0.89 - 1.20).

**Conclusions:**

Adjusting for the severity of the condition and adequate lactate clearance, cumulative fluid balance was not associated with increased mortality in this Latin American cohort of septic patients.

## Abstract (pt)

**Objetivo:**

Avaliar se a sobrecarga de fluidos na terapia hídrica é fator prognóstico para pacientes com choque séptico quando ajustada para os alvos de depuração de lactato.

**Métodos:**

Este estudo envolveu uma coorte retrospectiva e foi conduzido em um hospital de cuidados nível IV localizado em Bogotá, na Colômbia. Foi organizada uma coorte de pacientes com choque séptico, e suas características e balanço hídrico foram documentados. Os pacientes foram estratificados por níveis de exposição segundo a magnitude da sobrecarga de fluidos por peso corporal após 24 horas de terapia. A mortalidade foi determinada aos 30 dias, e foi desenvolvido um modelo de regressão logística incondicional com ajuste para fatores de confusão. A significância estatística foi estabelecida com nível de p ≤ 0,05.

**Resultados:**

Foram 213 pacientes com choque séptico e, após o tratamento, 60,8% deles tiveram depuração de lactato acima de 50%. Dentre os pacientes 97 (46%) desenvolveram sobrecarga de fluidos ≥ 5%, e apenas 30 (13%) desenvolveram sobrecarga ≥ 10%. Pacientes com sobrecarga de fluidos ≥ 5% receberam, em média, 6.227mL de soluções cristaloides (DP ± 5.838mL) em 24 horas, enquanto os não expostos receberam 3.978mL (DP ± 3.728mL), com p = 0.000. Os pacientes que desenvolveram sobrecarga de fluidos foram mais frequentemente tratados com ventilação mecânica (70,7% versus 50,8%; p = 0,003), albumina (74,7% versus 55,2%; p = 0,003) e corticosteroides (53,5% versus 35,0%; p = 0,006) do que os que não desenvolveram sobrecarga de fluidos. Em análise multivariada, o balanço acumulado de fluidos não se associou com mortalidade (RC 1,03; IC95% 0,89 - 1,20).

**Conclusão:**

Após ajuste para severidade da condição e depuração adequada de lactato, a ocorrência de balanço hídrico positivo não se associou com aumento da mortalidade nessa coorte latino-americana de pacientes sépticos.

## INTRODUCTION

Sepsis is one of the most frequent clinical syndromes that we face in critical care medicine. On average, the cause of admission of 20% of patients admitted to general intensive care units (ICUs) in the world is sepsis.^(1-5)^ Sepsis is a nonhomeostatic inflammatory response to infection that ends in organ damage. Furthermore, the progression to the development of septic shock is usually very serious, with a mortality of approximately 40.^(5-9)^ Global strategies have been put in place since 1991 around the world for the generation of clinical practice guidelines that make it possible to improve the results in the detection and treatment of this condition. The most well-known campaign is called Surviving Sepsis, which in its latest edition highlights the need for early detection, the control of the focus of infection, the administration of the right antibiotics and early goal-directed therapy (EGDT).^(10,11)^ This initial resuscitation is essentially based on the administration of isotonic crystalloid fluids, which have the highest level of evidence.^(11,12)^ Since then, fluid administration has become universal, for which the recommendation is to use 30mL/kg in the first 90 minutes and then as needed by the patient based on the presumption of response to the fluids.^(13)^ The problem with fluid administration as the first resuscitation measure is that its effect is erratic, unpredictable and very short-lived. This means that only 50% of patients truly respond to the administration of fluids, and therefore, with a high frequency, patients with sepsis develop fluid overload.^(14,15)^

Fluid overload in patients with septic shock is an almost universal finding; 70% of patients under strategies such as EGDT exhibit it within the first 24 hours, and almost half of these patients still have it on the third day.^(16,17)^ The development of volume overload is multifactorial and depends not only on the frequent administration of fluids but also on the presence of acute renal injury and the presence of capillary leakage associated with the inflammatory response in sepsis. Observational studies and post hoc analyses of clinical trials now show an association towards an increase in mortality rates to the extent that fluid overload occurs.^(18,19)^ However, this is not proof of even a causal relationship. In fact, it is the most critically ill patients who are more likely to receive more fluids during treatment because they are who most often lack therapeutic goals.

Therefore, our study aims to assess the association between the amount of resuscitation fluids in the first 24 hours of septic shock and the occurrence of death, adjusting for the presence of very important confounders such as the severity of the condition and the achievement of therapeutic goals, such as adequate lactate clearance.

## METHODS

The *Hospital Universitario San Ignacio* is a level IV hospital located in the city of Bogotá that serves the general population of the Colombian social security health system. The ICU has 25 beds and an ongoing registration of patients with significant pathologies, including sepsis. Based on these data, we explored the records for 2016, in which all patients diagnosed with sepsis were identified. The inclusion criteria for the cohort were being at least 18 years old and being diagnosed with septic shock based on the Third International Consensus Definitions for Sepsis and Septic Shock (SEPSIS 3) definition upon admission to the ICU.^(10)^ The exclusion criteria were pregnant women or women in their postpartum period; patients with cardiogenic shock, therapeutic limitation upon admission to the unit, errors in clinical history, confirmed diagnosis of Child-Pugh class B or C cirrhosis due to a family history, or shock due to recent spinal trauma; or patients in shock who died within 24 hours of admission. The study protocol was approved by the Ethics Committee at *Pontificia Universidad Javeriana*, which was the coordinating center.

### Exposure measurement

The level of exposure was determined through the review of the records of the fluids administered and eliminated upon the diagnosis of septic shock. The patients were stratified into various exposure levels, for which we took the fluids administered within 24 hours and the balance resulting from the algebraic sum of fluids administered minus those eliminated. Initially, patients with a fluid retention greater than 5% of their body weight were considered to be exposed to fluid overload. Individuals with lower balances were considered unexposed. However, given that the literature is unclear as to the best cut-off point for the presence of fluid overload, cut-off points at 7.5% and 10% of body weight were also assessed.^(20,21)^ Crystalloid solutions, in addition to fluids administered in blood and plasma components, were taken into account to assess the volume of fluid resuscitation.

### Outcome and definitions

The primary outcome was measured at 30 days as death from all causes as of admission to the cohort. The diagnosis of septic shock was based on the SEPSIS 3 definition.^(1,3)^ Patients with proven or probable infection who had vasopressor support to maintain a mean arterial pressure ≥ 65mmHg and a lactate level ≥ 2.0 were considered to be in septic shock. Cardiogenic shock was considered an exclusion criterion and is defined as the presence of systolic blood pressure < 90mmHg for more than 30 minutes or the requirement for vasopressor support to maintain normal values plus the presence of pulmonary congestion due to high filling pressures and signs of hypoperfusion or organ damage.^(22)^ The EGDT strategy was not used to establish the achievement of therapeutic goals. At present, there is sufficient evidence on the equivalence of other general measures of treatment.^(23,24)^ For this reason, patients who were able to reach and maintain a mean arterial pressure ≥ 65mmHg, an arterial saturation ≥ 90% and a urinary output ≥ 0.5mL/kg/hour in the first 6 hours were considered to be within the goals. In addition, having an arterial or central venous lactate level of less than 2mmol/L or a reduction of at least 50% of its initial value in the first 12 to 24 hours of therapy was considered a goal.

### Statistical analysis

With a significance of 0.05, a beta error of 0.2 and an expected mortality of 30% in patients without fluid overload, having a target odds ratio (OR) of 2.5, the minimum sample size was estimated as 180 patients using the Kelsey formula.^(25)^ Approximately 60 deaths were expected with a mortality of 30%. For this reason, at least 6 variables^( )^were adjusted to apply logistic regression.^(26)^ The database was built using the 2016 Excel package, while the analysis was performed with STATA 12.0. Patients were divided into exposed and unexposed patients. A description of the clinical variables is provided for each group. Qualitative variables are summarized as percentages, and quantitative variables are summarized as the means, medians with interquartile ranges (IQRs) and standard deviations (SDs).^(27)^ Comparisons between groups were performed using Student’s t-test or a Wilcoxon rank sum test depending on normality. The χ^(2 )^or Fisher’s exact test was used for qualitative variables according to the expected values. The level of significance was determined with a two-tailed test at p ≤ 0.05.^(26,27)^ The multivariable analysis was conducted through the construction of an unconditional logistic regression. The variables were selected through the so-called purposeful selection method (Hosmer & Lesmeshow, 2008). The drafting of the initial model included all variables and the set of multiplicative interactions. The final model or main effect model was obtained through a “backward” modeling strategy. Variables with a weak association with the outcome were excluded (p ≤ 0.20) using the Wald test.^(28,29)^ The presence of confounders was considered in modifications greater than 20% of the OR.

## RESULTS

**Figure 1:**
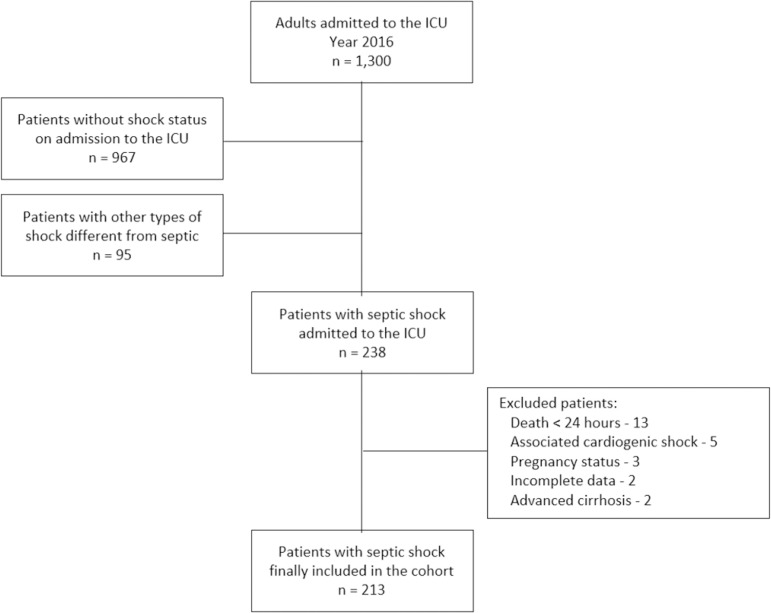
Enrollment in the study and cohort assembly. ICU - intensive care unit.

During the period between December 1, 2015, and December 30, 2016, 1300 adult patients were admitted to the ICU. Of these patients, 333 (25.6%) were admitted for treatment with a primary diagnosis of shock. Of the patients with a diagnosis of shock, 238 (71%) met the criteria for septic shock, 54 (16%) met the criteria for hypovolemic shock, 29 (8%) met the criteria for cardiogenic shock and 8 (2%) met the criteria for obstructive shock. All patients with a diagnosis of septic shock were included in the cohort, and there were no losses during follow-up. However, 25 (10.5%) were excluded for multiple reasons, the most frequent being early mortality within 24 hours following the start of therapy. The details of the construction of the cohort and the other causes of exclusion are provided in figure 1.

The final cohort was made up of 213 individuals, of which 131 (61%) were men; the average age was 58 years (SD ± 17.1), and the average body weight was 61.9kg (SD ± 12.5). Regarding the source of admission, 161 (75%) patients came from the emergency room, and the average time of treatment in resuscitation rooms was 14 hours (SD ± 11.7). The most frequent comorbidity of the cohort was cancer in 73 (34%) individuals, followed by systemic arterial hypertension in 71 (33%) individuals and diabetes mellitus in 36 (17%) individuals. Regarding the causes of septic shock, 129 (60%) individuals had positive cultures, and the most frequent foci of infection were the abdomen, with 57 (26.7%) cases, followed by the lung with 55 (25.8%) cases, 44 (20.6%) cases of bacteremia and 31 (14.5%) cases of urinary infection. Regarding the severity of the condition, the median Sequential Organ Faiulure Assessment (SOFA) score at the time of admission was 9 (IQR 7 to 12), 128 (60%) individuals received mechanical ventilation, 37 (17%) received continuous renal replacement therapy and 137 (64%) received colloidal fluids (albumin 20%). Regarding fluid therapy, the average resuscitation fluid volume used in the first 6 hours of therapy was 30.8mL/kg (SD ± 19.9), and the average fluid volume received in 24 hours was 82.7mL/kg (SD ± 34.1). In terms of the achievement of therapeutic goals, 60% of the cohort achieved lactate clearance ≥ 50% within 24 hours of therapy. As a result of the therapeutic process, 46% developed fluid overload ≥ 5% of their body weight in 24 hours, while only 13% developed fluid overload ≥ 10%. The 30-day mortality rate was 44% in 94 individuals (95% confidence interval - 95%CI 37 - 50).

Based on a fluid overload ≥ 5% of body weight within 24 hours, 114 individuals were stratified as unexposed patients and 99 as exposed patients. The percentage of men among exposed patients was 63%, compared to 59% among unexposed patients; the average age of exposed patients was 60.1 years, compared to 57.6 among unexposed patients; and the average body weight among exposed patients was 62.9kg, compared to 60.7kg among unexposed patients. No statistically significant differences were found in the comorbidities at the time of admission. However, the percentage of individuals with systemic arterial hypertension was strikingly higher, with 37.7% among unexposed patients *versus* 28.2% among exposed patients. Regarding laboratory tests conducted at the time of admission, there were no clinically relevant differences in the hemoglobin concentration or the platelet count. Differences were found in creatinine levels, with 1.69mg/dL among unexposed patients *versus* 2.32mg/dL among exposed patients, and in initial lactate levels, with 2.8mmol/L among unexposed patients *versus* 4.0mmol/L among exposed patients (these differences were also statistically significant, p < 0.05). Regarding the severity markers, the median SOFA score at the time of admission in exposed patients was 10, compared to 8 among unexposed patients. A summary of all comparative characteristics is provided in table 1.

**Table 1:** Comparison of clinical and socio-demographic characteristics between exposed and non-exposed patients

	Non exposed (n = 114)	Exposed* (n = 99)	Total (n = 213)
Men (%)	72 (63.2)	59 (59.6)	131 (61.5)
Age (mean ± SD)	60.1 (17.0)	57.6 (17.2)	58.9 (17.1)
Weight (Kg) (mean ± SD)	62.9 (11.9)	60.7 (13.0)	61.9 (12.5)
Arterial hypertension (%)	43 (37.7)	28 (28.2)	71 (33.3)
Diabetes mellitus (%)	19 (16.6)	17 (17.1)	36 (16.9)
Neoplastic disease (%)	40 (35.0)	33 (33.3)	73 (34.2)
HIV infection (%)	4.0 (3.5)	6.0 (6.0)	10 (4.69)
CKD (GFR < 60mL/min) (mean ± SD)	60 (52.6)	58 (58.5)	118 (55.4)
CKD 5 (Chronic dialysis) (%)	4 (3.5)	4 (4.0)	8 (3.76)
Hemoglobin (gr/dL) (mean ± SD)	10.8 (2.9)	10.7 (2.8)	10.8 (2.8)
Platelet count (103/uL) (mean ± SD)	209 (156)	197 (165)	203 (160)
Creatinine level (mg/dL) (mean ± SD)	1.69 (2.0)	2.32 (2.5)	1.98 (2.3)
GFR average (mL/min) (mean ± SD)	62.9 (36.2)	52.7 (35.6)	58.2 (36.2)
Lactate on admission (mmol/L) (mean ± SD)	2.8 (2.2)	4.0 (2.6)	3.42 (2.4)
Positive isolation (%)	72 (63.1)	57 (57.5)	129 (60.5)
Resistant germ on isolation (%)	37 (51.3)	27 (47.3)	64 (49.6)
Mechanic ventilation (%)	58 (50.8)	70 (70.7)	128 (60.0)
Continuous renal replacement therapy (%)	15 (13.1)	22 (22.2)	37 (17.3)
Hypertonic saline solution 3% (%)	29 (25.4)	35 (35.3)	64 (30.0)
Use of hypetonic albumin 20% (%)	63 (55.2)	74 (74.7)	137 (64.3)
Use of glucocorticoid (%)	40 (35.0)	53 (53.5)	93 (43.6)
SOFA score (median (IQR))	8 (7 - 10)	10 (7 - 13)	9 (7 - 12)
Lactate clearance > 50% (%)	77 (67.5)	52 (53.0)	129 (60.8)
Free days, mechanical ventilation (mean ± SD)	3.3 (3.3)	2.1 (2.4)	2.7 (3.0)
Days of hospitalization in ICU (mean ± SD)	6.4 (6.4)	5.6 (6.0)	6.0 (6.2)
30-day mortality from ICU admission (%)	40 (35.0)	54 (54.5)	94 (44)

SD - standard deviation; HIV - human immunodeficiency virus; CKD - chronic kidney disease; GFR - glomerular filtration rate; SOFA - Sequential Organ Failure Assessment; ICU - intensive care unit; IQR - interquartile range.

* Exposed, individuals with volume overload ≥ 5% of body weight at 24 hours.

**Figure 2:**
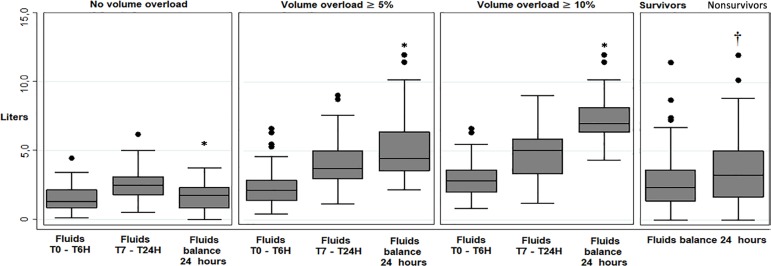
Comparison of the results of fluid therapy between exposed and unexposed patients. T0 - T6H - first 6 hours of therapy; T7 - T24H - therapy between 7 and 24 hours.

Regarding treatment, unexposed patients were resuscitated on average with 24.2mL/kg (SD ± 14.3mL/kg) in the first 6 hours compared to 38.4mL/kg (SD ± 22.3mL/kg) among exposed patients. Likewise, the average balance in 24 hours was positive, with 1620mL (SD ± 931mL) among unexposed patients compared to 5111mL (SD ± 2087mL) among exposed patients (Figure 2). In addition, 67.5% of unexposed patients obtained a lactate clearance ≥ 50% within 24 hours, compared to 57.0% of exposed patients. Individuals with fluid overload were more frequently treated with mechanical ventilation (70.7% *versus* 50.8%), received 20% albumin therapy more frequently (74.7% *versus* 55.2%), had a tendency towards a greater need for renal replacement therapy (22.2% *versus* 13.1%), and their mortality levels were higher, with 54.5% compared to 35.0% among unexposed patients (p = 0.004).

*Comparison between non-volume overload with volume overload 5% and 10%, p value < 0.05; †Comparison between survivors and nonsurvivors, p value < 0.05.

**Figure 3:**
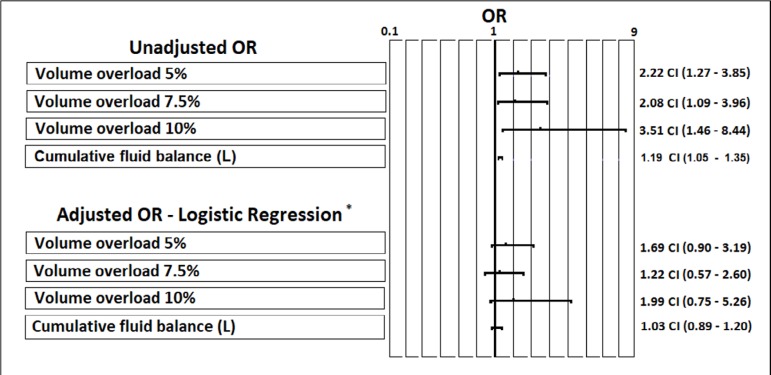
Logistic regression evaluating the relationship between volume overload and the development of death. OR - odds ratio. * The final model adjusted for the presence of cancer, Sequential Organ Failure Assessment score and the achievement of resuscitation goals.

Finally, the crude analysis regarding the occurrence of death and the cumulative fluid balance measured in liters within 24 hours initially showed a positive association, with an OR of 1.19 (95%CI 1.05 - 1.35). However, after adjusting for confounders, this association was not statistically significant, with an adjusted OR of 1.03 (95%CI 0.89 - 1.20). In the multivariable analysis, the variables that significantly predicted the occurrence of death were a family history of cancer, with an adjusted OR of 1.92 (95%CI 1.007 - 3.69), and the SOFA score, with an adjusted OR of 1.14 (95%CI 1.04 - 1.26). The achievement of the required lactate clearance was a protective factor, with an adjusted OR of 0.24 (95%CI 0.12 - 0.45). When using cut-off points related to the occurrence of fluid overload of 5.0%, 7.5% and 10%, there were no significant associations with the occurrence of death when adjusting for severity indexes, comorbidities or achieving adequate lactate clearance (Figure 3).

## DISCUSSION

Fluid overload in critically ill patients is a multifactorial phenomenon resulting from the interaction of factors including capillary leak syndrome, the common administration of fluids as a basic resuscitation therapy and hydrosaline retention associated with the frequent occurrence of kidney injury.^(16)^ This overload is considered to be deleterious and is frequently associated with higher mortality rates as well as more invasive procedures.^(16,20)^ Furthermore, the state of tissue perfusion and circulation in the presence of edema is made much more complex and could perpetuate circulatory dysfunction in addition to worsening the function of organs.^(30,31)^ However, it is clear that the risk of fluid overload also increases as the individual worsens and does not achieve his or her treatment goals, which in turn is associated with higher mortality rates.^(32,33)^ For this reason, fluid overload seems to be a severity marker and not necessarily a factor of poor independent prognosis. This relationship between the severity of the condition and the occurrence of fluid overload is much more consistent in the literature,^(34)^ and our research shows once again that these patients are more severely ill. In our cohort, patients with fluid overload showed a greater spectrum of severity than patients without fluid overload due to factors such as their creatinine level (2.32mg/dL *versus* 1.69mg/dL, p = 0.040), their lactate levels at the time of admission (4.0mmol/L *versus* 2.8mmol/L, p = 0.001) and their SOFA score (median 10 *versus* 8, p = 0.005).

In assessing the relationship between cumulative fluid balance and mortality, we did not find any relationship in our cohort when adjusting for important factors, such as the severity of the condition and the achievement of treatment goals (adjusted OR 1.03, 95%CI 0.89 - 1.20), or when using cut-off points in volume overload, such as 5.0%, 7.5% and 10% of body weight (Figure 3). In addition, the only factors that were able to independently predict the occurrence of death were the increase in the SOFA score (adjusted OR 1.14, 95%CI 1.04 - 1.26), a history of cancer (adjusted OR 1.92; 95%CI 1,007 - 3.69) and the achievement of a therapeutic goal in lactate clearance as a protective factor (OR 0.24, 95%CI 0.12 - 0.45). In our opinion, these findings support the fact that more severely ill patients more frequently develop fluid overload. However, if therapeutic efforts are able to lead the individual towards the achievement of treatment goals, fluid overload does not appear to be significant in the prediction mortality. The individual’s survival will ultimately depend on the balance between the severity of their condition and the achievement of therapeutic goals, where, in this case, achieving a reduction of lactate levels of more than 50% in 24 hours is a great protective factor. This does not necessarily justify the use of liberal fluid therapies, since it is clear that these patients require more days under mechanical ventilation and invasive treatments.^(35,36)^ However, these results show that therapy must always be adjusted to the actual treatment needs, such as the achievement of resuscitation goals. In fact, a recent study on the incorporation of a therapeutic bundle in sepsis showed that the application of simple measures such as early identification, rapid antibiotic administration and the administration of 30mL/kg of crystalloids are effective measures and are associated with a reduction in mortality and were not associated with adverse outcomes even in patients with a history of heart failure.^(37)^ This highlights that the measured administration of early fluids is probably justified, but it is necessary to quickly identify patients who do not respond to volume and therefore will not have an additional benefit of therapy. In fact, in our cohort, most of the volume overload in patients was acquired after the first 6 hours of therapy (Figure 2), which also draws attention to whether the liquids used in the treatment after the resuscitation phase are truly contributing to achieving therapeutic goals or simply represent the continuous administration of unnecessary solutions.

In the literature, the results are consistent with what was found in our study. If we consider cohort studies, there is agreement that patients with fluid overload are more severely ill.^(21,36,38)^ In the study conducted by Kelm et al.,^(18)^ which included septic patients using a clinical definition of the presence of edema, patients with fluid overload were affected by chronic kidney disease more frequently and had higher mortality scores than patients without fluid overload. In the study conducted by Sakr et al.,^(21)^ which also included only septic patients and used different fluid overload cut-off points based on fluid balance quartiles within 24 hours, the SOFA score increased with each increase in the water retention quartile. Likewise, other studies show a trend towards greater severity in patients suffering from fluid overload.^(36,39)^ However, establishing whether fluid overload is an independent prognostic factor is a much more complex task (and, in this regard, we have results that are difficult to interpret), and observational studies have deficiencies. First, regarding the definition of fluid overload, we found some studies with definitions that were based on clinical findings, other studies with definitions based on cut-off points at different moments in time and finally studies that compared only the highest quartiles of the balance with the most restrictive quartiles.^(21)^ Regarding specific research focusing on septic shock, the work conducted by Kelm et al.^(18)^showed that overload was able to independently predict mortality among septic patients (adjusted OR = 2.2, 95%CI 1.31 - 4.09) but failed to clarify whether the definition of fluid overload used would classify patients well due to greater water retention levels. In fact, there were no differences in the balance between patients with and without overload for days 1 and 3 of treatment. In contrast, the work conducted by Sakr et al.,^(21)^ which classified patients by balance quartiles in 24 hours, failed to demonstrate an association with the occurrence of death, even in the highest quartile (average balance of 5398mL in 24 hours), when adjusting for severity (adjusted OR = 1.07, 95%CI 0.76 - 1.51). In 2014, a systematic review of the literature on topics including all types of critically ill patients showed that restrictive fluid therapies were associated with lower mortality rates (OR = 0.42, 95%CI 0.32 - 0.55).^(20)^ However, this review is of very poor quality, since it combined patients from randomized clinical trials with those from observational studies, including a series of cases, which makes it impossible to control the presence of imbalances between groups, and it showed a very high degree of heterogeneity (I^(2 )^= 86%). In the end, we have the only large randomized clinical trial aimed at comparing restrictive and liberal fluid therapies in the therapeutic process. This trial was conducted with patients diagnosed with acute respiratory distress syndrome (ARDS), where approximately 60% were septic.^(35)^ Although patients on the restrictive side achieved a negative balance on day 7 compared to an average positive balance of almost 7L in the control group, there was no difference in mortality between the groups. This is the strongest evidence to date that supports the fact that fluid overload is not necessarily associated with higher mortality levels. Again, this does not support the routine use of restrictive liquid strategies, which can even be harmful if patients do not receive the appropriate amount of liquids in the first hours of resuscitation. Works such as that of Leisman et al. demonstrate that patients with chronic kidney disease and heart disease receive much less fluid in the early stages of resuscitation (120 minutes) and even in much later stages, which is typically associated with worse outcomes.^(40,41)^

Our research has strengths: the research is a cohort study primarily designed to search for an association; patients were selected consecutively; the study used SEPSIS 3 definitions; and, in particular, the study is one of the first works that adjusts for confounders in the achievement of therapeutic goals such as lactate clearance. Our research also has its weaknesses: the collection of patients was performed retrospectively, which increases the probability of measurement bias; the study was conducted at a single center; and it was not possible to specifically evaluate the EGDT goals within 6 hours of therapy (the lack of venous saturation or central venous pressure measurements within 6 hours). We believe there may be measurement bias in the patients’ body weight since the quality of the records in many of them made it impossible to verify the way in which patients were weighed. Finally, the use of arbitrary cut-off points in the definition of volume overload also made the precision in the OR calculations inadequate, for which a larger sample size is required. By virtue of the above, we believe that much more research is still needed on the subject, and a randomized clinical trial that directly tests the results of the use of restrictive fluid strategies in patients with septic shock is required. To date, the evidence is stronger towards outcomes such as the number of days with mechanical ventilation and the need for renal replacement therapies, which is no less important.^(16,20)^ However, regarding the prediction of mortality as an independent factor, we believe that fluid overload is just another marker of the severity of the patient’s condition and that the administration of liquids should be balanced, favoring early resuscitation without restrictions at 30mL/kg and after the first 3 hours establishing the true response of patients to volume.

## CONCLUSION

Adjusting for severity and adequate lactate clearance, cumulative fluid balance was not associated with increased mortality in this Latin American cohort of septic patients.
